# Distribution and functional significance of KLF15 in mouse cerebellum

**DOI:** 10.1186/s13041-025-01172-3

**Published:** 2025-01-21

**Authors:** Dan Li, Shuijing Cao, Yanrong Chen, Yueyan Liu, Kugeng Huo, Zhuangqi Shi, Shuxin Han, Liecheng Wang

**Affiliations:** 1 Department of Physiology, School of Basic Medical Sciences, Anhui Medical University, Hefei, Anhui 230032 China; 2 Department of Basic Medicine, Department of Clinical Medicine, West Anhui Health Vocational College, Liuan, Anhui 237000 China; 3 Cyagen Biosciences (Guangzhou) Inc., Guangzhou, Guangdong 510663 China; 4 Xinjiang Key Laboratory of Biological Resources and Genetic Engineering, College of Life Science and Technology, Xinjiang University, Urumqi, Xinjiang 830017 China

**Keywords:** KLF15, Transcription factor, Cerebellum, Purkinje cells, Neural development, Ataxia, Neurotransmitters, Gait analysis

## Abstract

Kruppel-like factor 15 (KLF15), a member of the KLF family, is closely involved in many biological processes. However, the mechanism by which KLF15 regulates neural development is still unclear. Considering the complexity and importance of neural network development, in this study, we investigated the potent regulatory role of KLF15 in neural network development. KLF15 was detected highly expressed in the cerebellum and enriched in Purkinje cells, with a significant increase in KLF15 expression between 15 and 20 days of neural development. Knockdown of KLF15 led to loss of Purkinje cells and impaired motility in mice. Therefore, our study aims to elucidate the relationship between KLF15 and Purkinje cells in mice, may provide a new research idea for the developmental mechanism of the mouse cerebellum.

**Supplementary Information:**

The online version contains supplementary material available at 10.1186/s13041-025-01172-3.

## Introduction

The cerebellum is an important component of brain tissue and has a recognized role in controlling motor functions such as coordination, balance, posture and skilled movements. Accumulating evidence has suggested that the cerebellum also plays a crucial role in non-motor functions such as cognition and emotion [1]. Therefore, defects in the cerebellum can cause a series of diseases, such as ataxia, muscle tone disorders, tremors, schizophrenia, etc [2]. The cerebellum is composed of several types of neurons, including granule cells, Golgi cells, Lugaro cells and Purkinje cells, of which the Purkinje cells are responsible for the main calculations in the cerebellum [3]. Studies have shown that activities mediated by different neurotransmitters have complex roles in neuronal migration [4]. The most extensively studied neurotransmitter is glutamate [5], and it usually promotes nerve migration [6–9]. Abnormal secretion of γ-aminobutyrate (GABA) neurotransmitters often affects the balance ability of mice [10]. Therefore, normal secretion of neurotransmitters is crucial for normal development of the cerebellum and balance of the body. However, the main influencing factor for the correct arrangement of neurons into layers during neural development [11] and the functions of other neurotransmitters such as acetylcholine (ACh) and glycine are still unclear [12–19].

Kruppel-like factor (KLF) is a transcription factor family with three highly conserved zinc finger structures that can bind to GC and CACCC sequences in the target gene promoters to exert their effects [20, 21]. KLF15, one of KLF family members, is widely distributed in many tissues [22, 23], participate in the body’s metabolism, tumors, immunity, and development [20, 24, 25].

However, there is limited research on KLF15 in neural development, research on the nervous system has mainly focused on KLF6 and KLF7 [26]. Some studies [27, 28] have reported that KLF7 can improve the survival rate of transplanted cells and promote axonal regeneration in models of peripheral nerve injury or spinal cord injury. Furthermore, KLF6 and KLF7 are essential transcriptional regulatory factors for optic nerve axon regeneration. The literature contains a few research reports about KLF15 in the nervous system, which indicates that KLF15 can inhibit the growth of neuronal axons [29] and that it shows a gradually upregulated trend in the late stages of mouse embryonic neural development [30]. However, the role and importance of KLF15 as a potential key factor in neural development have not been reported. In the present study, we detected the expression levels of KLF15 in different brain regions and found that it was highly expressed in the cerebella of mice. The cerebellum is an important brain area for coordinating proprioceptive motor function and also participates in advanced neural activities, such as cognition, emotion, and language processing [31–33]. The development of the cerebellum is regulated by various signaling molecules both inside and outside the cells [34, 35]; they play an important regulatory role in neurogenesis, lobulation, and cell proliferation and differentiation within the cerebellum [36]. The development of the cerebellum is crucial to the body [37]. Therefore, we conducted a series of tests to determine whether KLF15 is involved in cerebellum development and affects the regulation of motor function.

## Results

### The expression of KLF15 is higher in the cerebellum versus the rest brain regions and is significantly induced in the later developmental stages of the brain

**Fig. 1:**
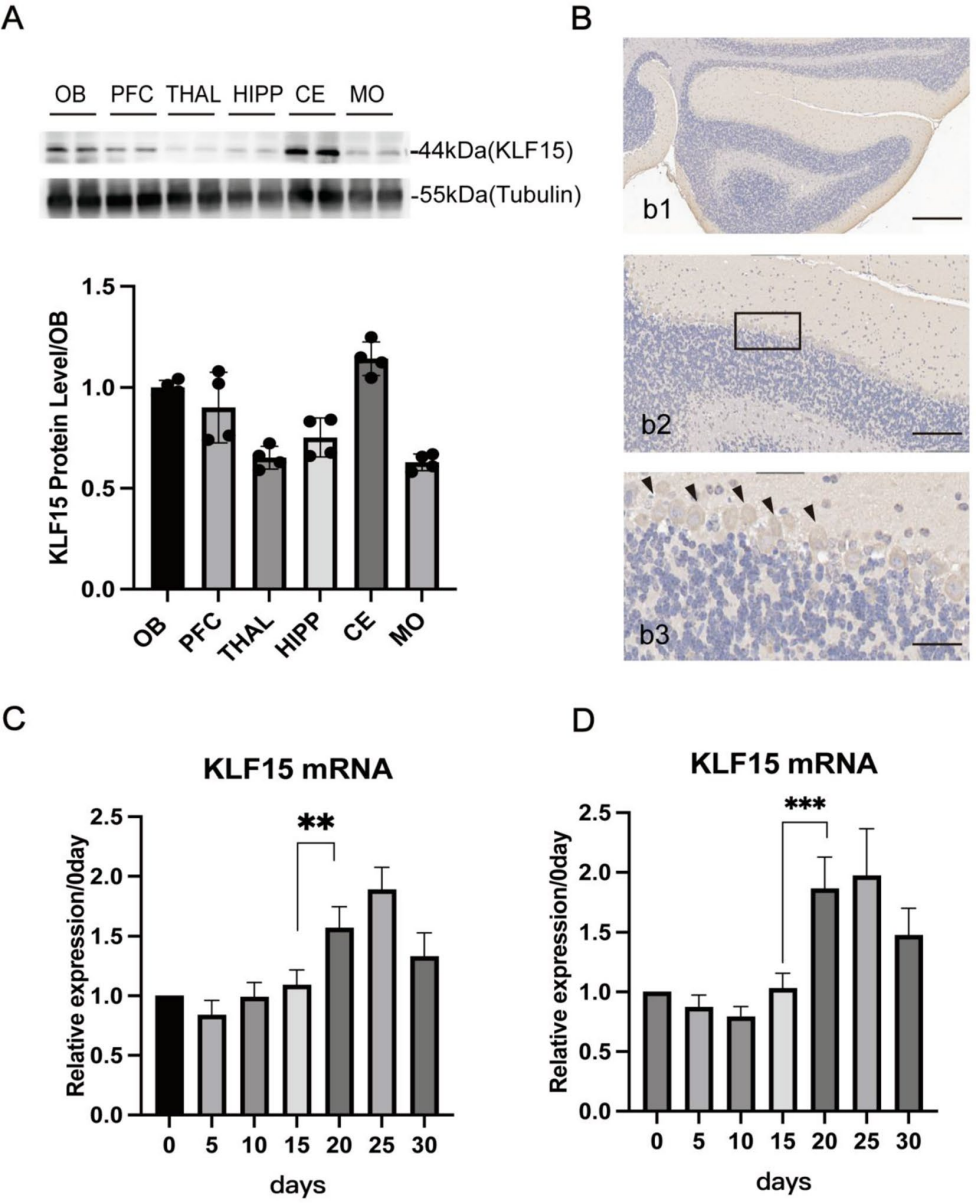
KLF15 is highly expressed in the cerebellum and significantly increases in the later stages of growth and development. **A** KLF15 is expressed in brain tissue with low tissue specificity, and its expression in the cerebellum is slightly higher than in other brain regions. (OB: olfactory bulb; PFC: prefrontal cortex; HIPP: hippocampus; THAL: thalamus; CE: cerebellum; MO: medulla oblongata). **B** KLF15 is highly expressed in Purkinje cells in the cerebellum. (b1: Scale bar = 200 μm; b2: Scale bar = 100 μm; b3: Scale bar = 50 μm). **C** The expression of KLF15 in the whole brain (excluding the cerebellum) at different ages (*n* = 5). **D** The expression of KLF15 in the cerebellum at different ages (*n* = 5). Data are shown as the mean ± SD. **P < 0.01, ***P < 0.001, vs. 15days (two-tailed Student’s t-test)

We first detected that KLF15 was expressed in most brain regions in 7-week-old mice, with a slightly higher expression level in the cerebellum (Fig. 1A). Further immunohistochemistry was performed on mouse cerebellum slices of 7-week-old mice, and the results showed that KLF15 was mainly located in the Purkinje cells (Fig. 1B). Finally, the mRNA expression of KLF15 in the whole mouse brain and cerebellar extracts was monitored at different developmental stages (Fig. 1C-D). Notably, a significant induction of KLF15 expression after day 15 in the later developmental stages. The results suggest that KLF15 may be involved in the division and differentiation of neural cells in the later stages of cerebellum development.

### Construction of viruses and verification of knockdown efficiency

**Fig. 2:**
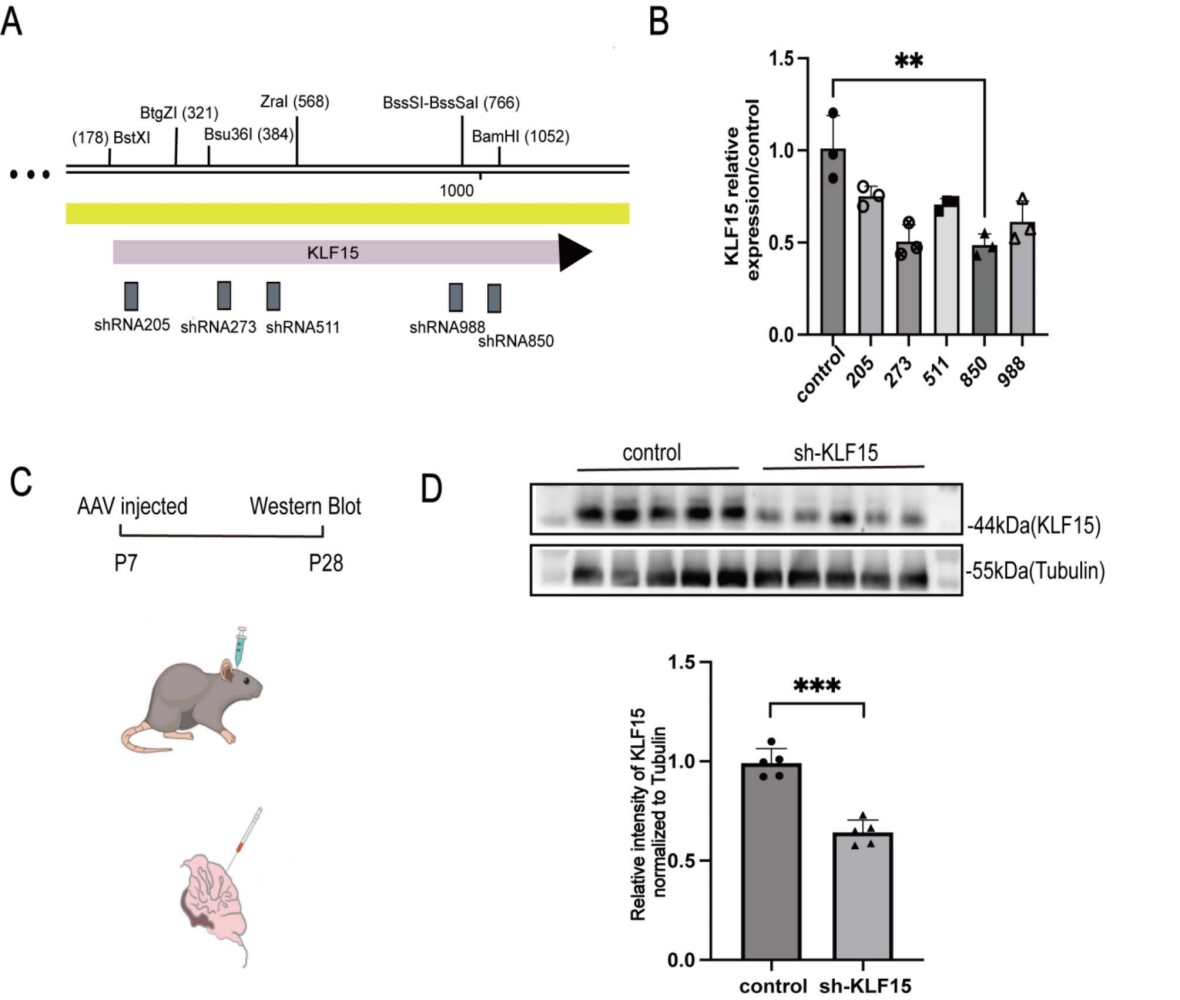
KLF15 knockdown virus can effectively knock down mouse cerebellar KLF15. **A** The positions of five interfering sites on the KLF15 gene. **B** Real time fluorescence quantitative PCR detection of plasmid knockdown efficiency at different target sites. **C** Sh-KLF15 virus stereotactic injection into the cerebellum. Target 850 site plasmid as the optimal knockdown target for virus packaging and cerebellum stereotactic injection. **D** Western blot verifies virus knockdown efficiency on the 21st day after injection. Data are shown as the mean ± SD. **P < 0.01, ***P < 0.001, vs. control group (two-tailed Student’s t-test)

In order to investigate the effect of KLF15 knockdown in vivo, we designed multiple RNA interference target sequences based on the target gene sequence and using the RNA interference sequence design principles provided on public websites. Based on our design experience and software, we conducted an evaluation and determination to select the optimal kinetic parameter target for the subsequent experimental process, we constructed knockdown plasmids with five different targets and screened for knockdown efficiency in alpha mouse liver 12 cells for adeno-associated virus (AAV) packaging. The five interference sites are shown in Fig. 2A. We found that the knockdown efficiency of the target 850 site was the best (Fig. 2B). Following this, we constructed an AAV9 strain virus (GPAAV-HU6-sh-KLF15-CMV-eGFP-WPRE. Titer, 5.41E + 12 vg/ml) and performed stereotactic injections into the cerebella of 7-day-old mice at coordinates x (M/L): 0 mm y (A/P): -3.7 mm z (D/V): -1.2 mm. Using 10 µl micro injection needle inject 350 nl of virus (Fig. 2C). At the same time, we injected virus containing the control plasmid (GPAAV-HU6-Scramble-CMV-eGFP-WPRE. Titer, 1E + 12 vg/ml) into the control mice. After 21 days of virus infection, KLF15 expression was tested to verify the efficiency of viral expression. The results showed a significant decrease in cerebellum KLF15 levels in the sh-KLF15 viral injection group compared to the control group (Fig. 2D). This indicates that virus transfection in the cerebellum of 7-day-old mice for 21 days effectively inhibited the expression of KLF15. In order to detect the specificity of AAV virus targeting KLF15, we tested the genes regulating Purkinje cell development Stxbp2 and Tanc1, ataxia related genes Ifrd1, Sptbn2, and Elovl5, genes regulating granule cell development Gap43 and Stmn2 [38], as well as other KLF family genes KLF7 and KLF13 that can regulate nerve cells. The results showed that AAV virus did not regulate the expression of these genes (Additional Fig. 1). However, due to the use of only one type of shRNA, we have to acknowledge the possibility of off target effects of sh-KLF15.

### Deficiency of cerebellum KLF15 during the growth stage leads to motor ataxia in mice

**Fig. 3:**
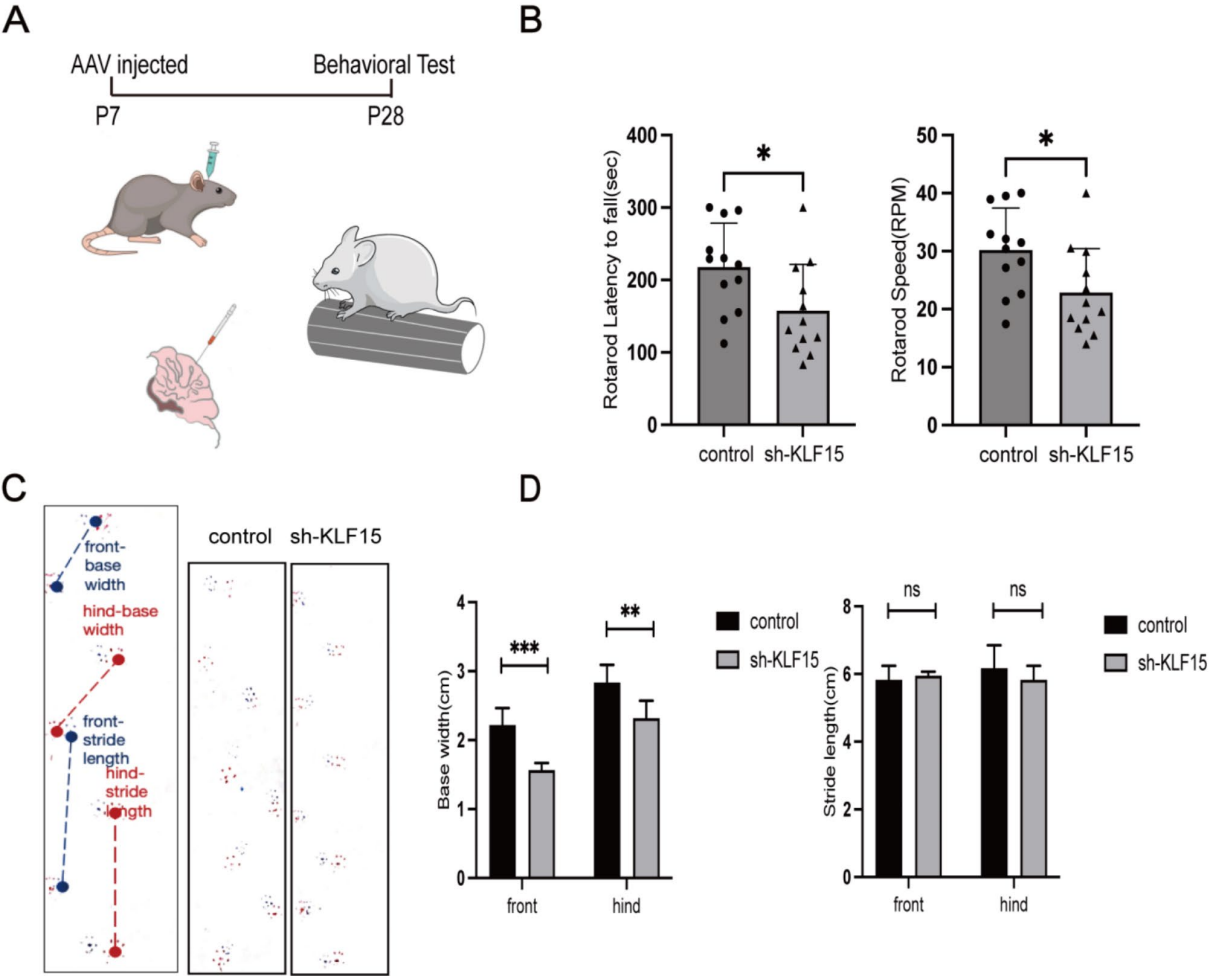
Impaired motor ability in KLF15 cerebellum knockdown mice. **A** Schematic diagram of 7-day-old cerebellum AAV microinjection (left). The rotarod test measures the balance and coordination ability of a rotating stick on the 21st day after the virus is infected (right). **B** Compared to the control group, knockdown mice showed a significant decrease in their time spent on the rod before falling. The rod speed at which KLF15 knockdown mice fall from the pole also yielded similar results. **C** Gait analysis. After 21 days of virus infection, mice were allowed to walk in a narrow 50 cm corridor with ink showing footprints. **D** Gait analysis statistical results. Compared to the control group, there was no significant difference in step length between the knockdown group and the control group, but the step width significantly narrowed (*n* = 6). Data are shown as the mean ± SD. *P < 0.05, **P < 0.01, ***P < 0.001, ns, no significant, vs. control group (two-tailed Student’s t-test)

To investigate the effect of KLF15 knockdown on motor function, we injected the AAV-shKLF15 virus (GPAAV-HU6-sh-KLF15-CMV-eGFP-WPRE) in the cerebella of the mice, and we conducted behavioral testing 21 days after injections (Fig. 3A, C). Rotarod tests showed that mice with KLF15 knockdown exhibited significant motor deficits compared to the mice in the control group, as demonstrated by a significant reduction in residence time on the rotating rod and the rate of rod rotation during drop in the knockdown group was significantly lower than that in the control group (Fig. 3B). A gait analysis showed that, compared to the control mice, the anterior and posterior basal widths of the KLF15 knockdown mice had significantly reduced (Fig. 3D). These results suggest that KLF15 knockdown in the cerebellum during the growth stage would lead to motor ataxia in mice.

### The reduction of KLF15 causes the loss of Purkinje cells in the cerebellum and abnormal dendritic morphology

**Fig. 4:**
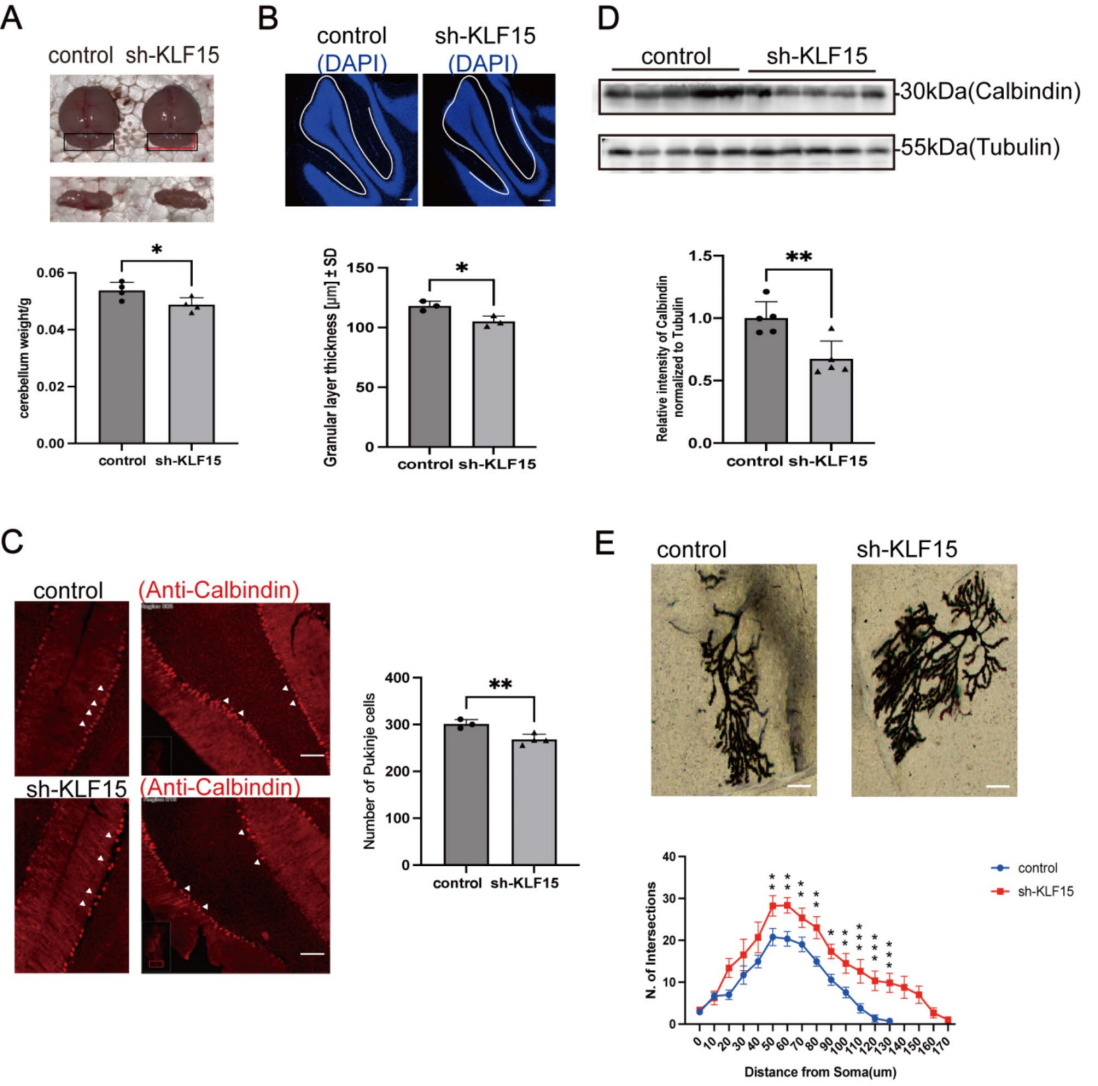
Loss and morphological abnormalities of Purkinje cells in the cerebellum of KLF15 knockdown mice. **A** Weighting the cerebellum of control mice and KLF15 knockdown mice. The cerebellum weight of the knockdown group was significantly lower than that of the control group. **B** Immunofluorescence staining of cerebellum in the control mice and sh-KLF15 mice. KLF15 knockdown mice have significantly thinner granular layer than control group mice. Scale bar = 200 μm. The white line represents the thickness of the granular layer in the control group. **C** Immunofluorescence staining of cerebellum in the control mice and sh-KLF15 mice. On the right is the quantitative statistics of Purkinje cell number. Scale bar = 200 μm. **D** Western blot and grayscale analysis of calbindin in the cerebellum of control mice and KLF15 knockdown mice. **E** Representative images of Golgi staining in the cerebellum of control mice and KLF15 knockdown mice. Scale bar = 80 μm. Sholl analysis images of Purkinje cells (*n* = 6). Data are shown as the mean ± SD. *P < 0.05, **P < 0.01, ***P < 0.001, vs. control group. Two-tailed Student’s t-test **A**,** B**,** C**,** D** and two-way ANOVA followed by Tukey’s test **E** were used for data comparison

To explore the possible role of KLF15 in mouse cerebellum, we knocked down KLF15 in the cerebella of 7-day-old mice by AAV-shKLF15. KLF15 knockdown led to decreased cerebellar weight and capacity (Fig. 4A). Afterwards, we performed immunofluorescence analysis on cerebellar lobes IV and V and found that the KLF15 knockdown group had a significantly thinner granular layer than the control group (Fig. 4B). Since KLF15 was found to be concentrated in Purkinje cells, we performed an immunofluorescence analysis of the calcium-binding protein, a marker of Purkinje cells in mice cerebella [39, 40]. The results showed a significant reduction in Purkinje cells in the knockdown mice (Fig. 4C). Western blot analysis further showed a significant decrease in the calbindin levels in the knockdown mice (Fig. 4D). The reduction in cerebellum weight and capacity suggests that KLF15 deficiency in the growth stage leads to neuronal loss. We did not find any changes in the number of Purkinje cells when knocking down KLF15 in the cerebellum of adult mice (Additional Fig. 2). These results suggest that KLF15 is an important regulatory factor for Purkinje cell during the growing stage of mice.

Dendritic morphology is closely related to neuronal function. Golgi staining was performed to investigate the dendritic morphology of Purkinje cells in the cerebella of KLF15 knockdown mice. The results showed that the dendritic complexity of Purkinje cells was higher in these knockdown mice than in the control group (Fig. 4E). Sholl analysis showed that knocking down KLF15 resulted in a significant increase in the intersection of dendrites with concentric circles centered on the cell body (Fig. 4E). Studies have shown that retinal ganglion cells containing KLF15 plasmids exhibit significantly shorter axon lengths than control cells, indicating that KLF15 is an inhibitory factor in neuronal development [29]. We also showed that KLF15 expression was significantly elevated in the late stage of neural development in mice (Fig. 1D). These results indicate that KLF15 is a regulatory factor that inhibits neuronal overgrowth and maintains a balance in the number of cerebellar neurons. Knocking down KLF15 disrupted this balance, resulting in abnormal numbers of neurons and abnormal dendritic morphology.

### KLF15 regulates the levels of neurotransmitters in the cerebellum

**Fig. 5:**
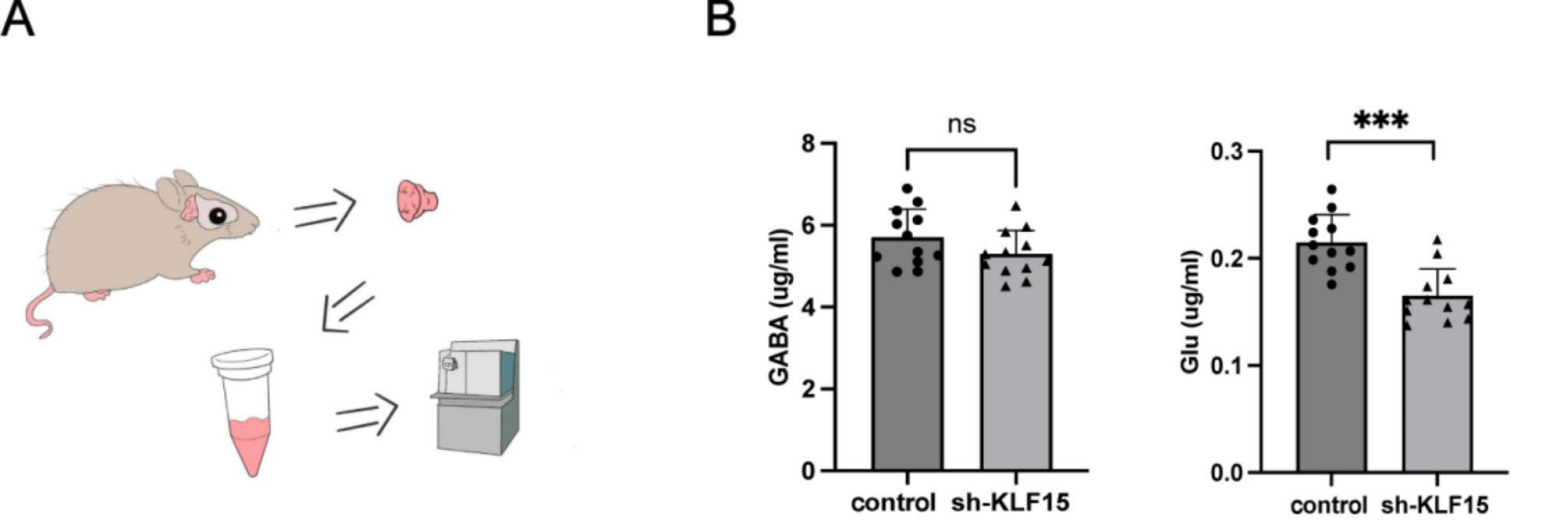
KLF15 knockdown mice display abnormal levels of cerebellum neurotransmitters. **A** High-performance liquid chromatography/mass spectra analysis of mouse cerebellum homogenate. **B** Compared to the control mice, knockdown mice showed no significant changes in the content of neurotransmitter GABA, but a significant decrease in the content of neurotransmitter Glu in the cerebellum. Data are shown as the mean ± SD. ***P < 0.001, ns, no significant, vs. control group (two-tailed Student’s t-test)

Glutamic acid (Glu) and γ-aminobutyrate (GABA) are important neurotransmitters that regulate body balance. To determine whether KLF15 affects the levels of these neurotransmitters, we used high-performance liquid chromatography–mass spectrometry to detect GABA and Glu contents in the cerebella of mice (Fig. 5A). We found no significant difference in GABA content but a significant reduction in Glu (Fig. 5B). We believe this may be due to the thinning of the granular layer thickness in KLF15 knockdown mice. The thinning of the granular layer implies a decrease in the number of granule cells, which may further affect the secretion of Glu and lead to a decrease in Glu content.

## Discussion

KLF15 is known to play important roles in many pathophysiological functions [41, 42]. However, there is limited investigations on the role of KLF15 in the nervous system except for a previous study indicating that KLF15 is possibly involved in the neural development [29]. Our current research reveals the effective regulation of cerebellar KLF15 on motor function and dendritic morphology of Purkinje cells. We found that KLF15 expression was higher in the cerebellum, especially the Purkinje cells, and knocking down cerebellar KLF15 resulted in the loss of Purkinje cells. Several transcription factors such as *Znf212* [39], *Smad2* [40], and *Tmem30a* [43] have been reported to be involved in the development of Purkinje cells. The absence of these transcription factors can cause abnormal cerebellar development and loss of Purkinje cells, ultimately leading to ataxia in mice. Besides, the loss of Purkinje cells may be caused by a series of changes in transcription factors, which may involve downstream gene imbalance leading to abnormal protein folding modification [44], activation of apoptosis [45], and an increase in nutritional neurons such as astrocytes [46]. In addition, the absence of transcription factors related to Purkinje cell development can also cause endoplasmic reticulum stress [47], abnormal expression of signaling proteins in related pathways [48], and a series of molecular changes. Our research on these mechanistic pathways is still insufficient, but our preliminary exploration of the specificity of KLF15 in the neural field is innovative. At the macro level, we first demonstrate the close relationship between KLF15 and Purkinje cell. The integrity of Purkinje cells is necessary for maintaining balance in the body, and these are the only neurons in the cerebellar cortex that emit output signals [49], so the normal functioning of these cells is crucial for maintaining body balance.

Studies have shown that normal levels of GABA/Glu are necessary for maintaining normal body movement [50]. We used high-performance liquid chromatography–mass spectrometry and found a decrease in Glu content. Studies have shown that the GABA neurotransmitter plays an important role in regulating neuronal excitation and that the disruption of GABA transmission can lead to seizures [51], whereas excessive Glu content can lead to excessive nerve excitement. In the early stages of neural development, GABA is the main inhibitory neurotransmitter in brain tissues. It depolarizes immature neurons and exerts a dual effect of excitation and shunt/inhibition in the developing neural network [52]. In recent years, there have been more studies on the neurotransmitters GABA and Glu related to emotions [53], such as the GABA pathway regulating anxiety like behavior in Parkinson’s rats [53] and the involvement of GABA/Glu in the occurrence of depressive emotions [54]; Related to brain metabolism [55], there is a significant correlation between GABA and low metabolism in the brain; Related to pain [56], such as brain-derived neurotrophic factor (BDNF) converting the normal inhibitory effect of GABA into stimulation, leading to the occurrence of neuropathic pain [57]. The balance between GABA and Glu affects synaptic plasticity and regulates chronic inflammatory pain [56]. Our research on neurotransmitters mainly aims to explain the changes in neurotransmitter content caused by knocking down KLF15, which is reflected in the correlation between the balance and coordination ability of GABA and Glu in the traditional sense. The detection of changes in GABA and Glu content is intuitive and can clearly indicate that the decrease in balance and coordination ability of mice is related to the decrease in Glu content. It is noteworthy that compared to control mice, there were no significant differences in GABA content in sh-KLF15 mice. We believe that cellular morphology changes are closely related to changes in neurotransmitter content. In the knockdown mice, the loss of Purkinje cells and the denser dendritic development of Purkinje cells may complement each other, resulting in no significant change in GABA content. However, the discrepancy between the Glu and GABA results is not yet clear and remains to be illustrated by further in-depth research.

There are few reports on KLF15 in the nervous system. In this study, our data for the first time demonstrates the specificity of KLF15 in the field of the nervous system. KLF15 is mainly distributed in the cerebellum and is expressed more abundantly in Purkinje cells. Subsequently, we elucidated the effects of KLF15 on the cerebellum of juvenile mice from several different perspectives. The conclusion is that KLF15 is crucial for maintaining the quantity and dendritic morphology of Purkinje cells. Knocking down KLF15 can lead to a decrease in the number of Purkinje cells, denser dendritic morphology, abnormal neurotransmitter levels, and impaired balance and coordination abilities.

Although our study provides some evidence for the function of KLF15 in the nervous system, there are still some limitations that need to be addressed in future research. Firstly, our research on the impact of KLF15 on the state of Purkinje cells is relatively superficial and does not involve specific mechanistic pathways. The development of Purkinje cells is inevitably regulated by multiple factors, so further exploration of the underlying mechanisms is necessary and meaningful. Secondly, further explanation of neurotransmitter content and subsequent experiments still lack relevant detection data, and the explanation of synaptic function is not yet comprehensive. These two limitations still require a significant amount of time to be explored in the future, which is beyond the scope of this study.

In summary, the findings of this study showed that KLF15 plays an important role in maintaining normal dendritic morphology of Purkinje cells, and the normal levels of neurotransmitters, thereby contributing to normal balance and coordination of mouse movement.

## Methods

### Animals

Approval for all experimental animals was provided by the Ethics Committee of Anhui Medical University. Ethical approval for this study (No. LLSC20190763) was provided by the Institutional Animal Care Unit Committee of Anhui Medical University on October 10, 2019. The C57BL/6 background mice were purchased from Henan Skobes Biotechnology Co., Ltd. and raised in polypropylene cages. They were subjected to a 12 h light–dark cycle (with the lights on at 8:00 a.m. and off at 8:00 p.m.), and the indoor temperature was maintained at around 26 °C. Adequate food and water were provided with free access.

### Western blotting

Radioimmunoprecipitation assay lysis buffer (RIPA) and protease phosphatase inhibitor were added to an organized EP tube and ground. The supernatant was centrifuged, 5× loading buffer was added, and the sample was boiled. Following this, polyacrylamide gel electrophoresis (SDS-PAGE) was prepared, the sample was added, and the protein was transferred onto the PVDF membrane for electrophoresis. After the membrane transfer is completed, seal with 5% skim milk for 2 h. Dilute 5% skim milk with TBST for preparation. After sealing, rinse with phosphate buffered saline (PBS) once. Prepare the primary antibody incubation solution using the dilution solution according to the recommended ratio in the instruction manual: rabbit anti-KLF15 (1:1000 dilution, Abcepta, #AP18136b); mouse anti-GAPDH (1:5000 dilution, Immunoway, #YM3029); rabbit anti-Calbindin (1:1000 dilution, Huabio, #ET1702-54). Incubate overnight on a shaking table at 4 ℃ for approximately 15 h. Wash the first antibody with TBST for 15 min each time, three times. (Due to the high cost of primary antibodies, they can be recycled and reused approximately three times). Dilute the secondary antibody corresponding to the primary antibody with 5% skim milk prepared with TBST. Prepare the secondary antibody incubation solution according to the recommended ratio in the instruction manual. Incubate at room temperature on a shaker for 2 h. Wash the secondary antibody with TBST for 15 min each time, three times. Exposure images were obtained by combining enhanced chemiluminescence (ECL).

### RT-qPCR

The required mouse was euthanized by cervical dislocation method, with the left hand pressing on the mouse’s head and the right hand gripping the end of the tail 1/3. The right hand was pulled back until a sense of emptiness was felt. After cervical dislocation in mice, the head is cut off, the head skin is cut open, the skull is T-shaped, the brain tissue is exposed, and the cerebellum is carefully peeled off. The detached cerebellum is rinsed in pre cooled PBS to remove blood stains and hair. It is then placed on filter paper to absorb surface moisture and placed in a labeled centrifuge tube for immediate use in the experiment. We used the traditional Trizol method to extract RNA from mouse cerebellum tissues. Detection of RNA concentration using NanoDrop-2000c ultra micro spectrophotometer. Single-stranded RNA was used to synthesize cDNA. According to the manufacturer’s instructions, two-step quantitative PCR was performed using the 2x SYBR green reagent and primers corresponding to the target gene to obtain its Ct value. The primers were synthesized from the official website of biotechnology (https://www.ncbi.nlm.nih.gov/ NCBI).

**Table:** 

Target genes	Primer sequence(5’-3’)
GAPDH	F: CATCACTGCCACCCAGAAGACTG R: ATGCCAGTGAGCTTCCCGTTCAG
KLF15	F: ACACCAAGAGCAGCCACCTCAA R: GCCTTGACAACTCATCTGAGCG
Stxbp2	F: GAACGCCTTCAAGGCAGACACT R: CATGGCTTGGAACGTGAGTTCG
Tanc1	F: TCTCGACAGGAGAGCAAGCTGA R: CAGATGGCTTGAGGAGACTCCA
Gap43	F: TGTGCCTGCTGCTGTCACTGAT R: AGGTTTGGCTTCGTCTACAGCG
Stmn2	F: CAGAAGCTCCACGAACTCTAGC R: TGCTTCAGCACCTGAGCCTCTT
Ifrd1	F: CTGGCGAATCTTTGGCACTTCTG R: ACCGCTGCTTTCTCTTGTCCAC
Sptbn2	F: GTGGCAGAAACACCAGGCATTC R: CTCCAGCTTCTCTGACACTACG
Elovl5	F: GGTGGCTGTTCTTCCAGATTGG R: CTTCAGGTGGTCTTTCCTCCGA
KLF7	F: GGAAGGATGCGAGTGGCGTTTT R: CGCAAGATGGTCAGACCTGGAG
KLF13	F: CAGAGGAAGCACAAGTGCCACT R: GCGAACTTCTTGTTGCACTCCTG

### Construction of plasmids

We linearized the PGMAAV-10,261 RNAi vector using restriction endonucleases, connected the target RNAi sequence, and constructed a vector with the target RNAi sequence. Target RNAi sequence:

**Table:** 

Target	Target RNAi sequence
NC	TTCTCCGAACGTGTCACGT
shRNA205	GACCTTCTCGTCACCGAAATG
shRNA511	GCCTGTGAAGGAGGAACATTT
shRNA988	GAACCTGCCCTCAAAGTTTGT
shRNA273	CAGTGGAGGTATTGGAGATAG
shRNA850	CCCATTGCCGCCAAACCTATT

### Stereotactic injection

For young mice, anesthetized with isoflurane, place the mice on a stereotaxic stand, insert an ear stick into the external ear canal of the mice, and fix the front teeth to keep the posterior fontanelle level. Due to the small size of the mice and the fact that their hair has not yet grown, we only disinfected the outer surface of the skull skin with 75% ethanol, cut open the scalp, and exposed it. After exposure, we used a dental drill to drill a small hole above the cerebellum at coordinates x (M/L): 0 mm y (A/P): -3.7 mm z (D/V): -1.2 mm. Using 10 µl micro injection needle inject 350 nl of virus (control virus: GPAAV-HU6-Scramble-CMV-eGFP-WPRE. Titer, 1E + 12 vg/ml; sh-KLF15 virus: GPAAV-HU6-sh-KLF15-CMV-eGFP-WPRE. Titer, 5.41E + 12 vg/ml. Genomeditech. ) into each site at a rate of 60 nl/min, and leave for 5 min after injection. After injection, the mice were placed on a heating pad to slowly awaken. Put it back into the mouse cage after 30 min. Follow up experiments will be conducted around 3 weeks after virus infection.

### High-performance liquid chromatography–mass spectrometry

After sampling, weigh it and add physiological saline in a ratio of 2 ml/g. Place two grinding beads in each EP tube and grind 5 times at a frequency of 60 Hz in a homogenizer, with a 60 s interval between each time. Put the ground homogenate into a 4 ℃ centrifuge at 12,000 ×*g* and centrifuge for 10 min. Take the upper clear 500 µl. Add an equal amount of acetonitrile, shake thoroughly and mix for 30 s. Centrifuge at 12,000 ×*g* at 4 ℃ for 10 min, and take 0.22 of the supernatant µm organic filter membrane. Take 450 µl. Join 50 µl GABA-D_6_ 1 × 10^− 7^ mg/ml internal standard, shake and mix well for later use. The experimental instrument is the Shimadzu UPLC system, and the chromatographic separation column is Shim pack GIST C18 column (2.0 μm. 2.1 mm × 100 mm). The mobile phase A is 0.15% methanol water, and the B phase is methanol. The temperature of the column temperature box is 40 ℃. The total flow rate is 0.2000 ml/min. After stabilizing the pump pressure, pump A has a pressure of 21.5 MPa and pump B has a pressure of 21.3 MPa. Injection volume of 10 µl. The gradient elution program is 0–6 min, 90% A; 6–9 min, 90% -10% A; 9–15 min, 10% A; 15–18 min, 10 -90% A; 18–23 min, 90% A.

### Immunohistochemistry

Anesthetized mice were intraperitoneally injected with pentobarbital (50 mg/kg), and 50 ml of physiological saline NaCl and 25 ml of paraformaldehyde PFA were perfused into the heart. The mouse brains were removed through craniotomy and incubated in polyformaldehyde (PFA) at 4 °C for 24 h. Then, sugar was gradually precipitated, sucrose solution was prepared with 20% phosphate buffered saline (PBS) for 24 h, and then with 30% PBS for 24 h for tissue dehydration. Using a frozen slicer, 30 μm brain slices were cut and then washed with a PBS embedding agent three times every 5 min. Prepare 6% donkey serum, 1% BSA, and 0.6% tramadol PBST using PBS and drop them onto brain slices, with approximately 50 µl drops per slice. Closed for 2 h. Dilute the primary antibody with the prepared PBST according to the instructions: goat anti-KLF15 (1:200 dilution, Abcam, #Ab2647), incubate on brain slices for 36 h, and wash the primary antibody. Dilute the secondary antibody with prepared PBST according to the instructions and incubate it on brain slices for 2 h. Wash the secondary antibody, shake off the secondary antibody, and wash with PBS three times for 5 min each time. Dry the glass slide at room temperature. Slowly cover the cover glass and apply nail polish on the four corners.

### Immunofluorescence

After obtaining the mice, anesthesia was administered with isopentane. After the mice became unconscious, they were placed on foam plates to fix their limbs, and the chest was cut open with scissors, exposing the heart. Insert a venous needle from the left apex of the heart and inject physiological saline. At this time, the heart will swell and the right atrial appendage will be cut open. Slowly inject 40 ml of physiological saline and 20 ml of polyformaldehyde PFA. Injecting paraformaldehyde found that the tail of the mouse was stiff, the limbs twitched, and the liver turned white, indicating a successful perfusion. The preprocessed brain slices were sealed with a PBST locking solution prepared with PBS, 6% donkey serum, 1% BSA, and 0.6% Triton X-100. The first antibody was diluted in proportion to PBST: rabbit anti-Calbindin (1:200 dilution, Huabio, #ET1702-54) and added dropwise onto the brain slices for overnight incubation at 4 °C. The fluorescent secondary antibody was combined with its corresponding species for 2 h. The nuclei were stained with 4,6-diamidino-2-phenylindole (DAPI) staining solution for 5 min. Following this, an anti-fluorescence quenching agent was added dropwise, and the film was sealed. The panoramic tissue cell quantitative analysis system TissueFAXSPlusS was used to obtain staining images.

### Golgi stain

Intraperitoneal injections of pentobarbital (50 mg/kg) were used to anesthetize the mice. 50 ml of physiological saline and 25 ml of paraformaldehyde PFA were perfused into the heart to obtain brain tissue samples, which were then soaked in Golgi fixative. After 48 h of fixation, the tissues were cut into blocks of 2–3 mm thickness based on the desired observation site. Golgi staining solution was added until the tissue blocks were completely immersed, which were then placed in a cool, ventilated place, avoiding light and reacting for 14 d. After washing with distilled water, the tissue blocks were immersed in 80% acetic acid and left to sit overnight. These were then transferred into 30% sucrose solution. A slicer was used to cut out 100 μm slices. These were air dried and treated with concentrated ammonia water for 15 min, washed with water for 1 min, treated with an acidic film fixing solution for 15 min, and sealed with glycerol gelatin. Panoramic images of the brain tissues were obtained using a digital slice scanner.

### Rotarod testing

The rotating rod experiment is used to measure the balance and coordination ability of mice. Three days before the official experiment, the mice were first subjected to adaptive training, gradually accelerating from 4 r/min to 40 r/min to adapt to the state of being on a rotating stick. The training lasted for three consecutive days. During the formal experiment, first maintain the rotating rod at 4 r/min, place the mouse on the rotating rod to adapt for 1 min, and then accelerate from 4 r/min to 40 r/min. Record the time of the mouse’s fall and the rate of rotation of the stick during the fall.

### Gait analysis

We use a flat track with a length of 50 cm and a width of 9 cm, with white paper laid on the bottom, and let the mice walk along the track. To visualize the footprints of mice, we applied blue ink to the front paws and red ink to the back paws. Carefully place the mouse at the beginning of the white paper and let it walk forward, leaving its footprints on the paper. After the ink dries, measure its step length and width with a ruler and record the data.

### Sholl analysis

We drew concentric circles with the cell body at the center, increasing the radius by 10 μm each time, and counted the number of intersection points between the cell dendrites and the concentric circles.

### Statistical analysis

The data for at least three independent experiments were expressed as mean ± SD values. A t-test or a two-way ANOVA followed by Tukey’s test was used to compare and analyze the statistical differences between two groups of data. GraphPadPrism8 was used for statistical analysis and chart production. * *p* < 0.05; ** *p* < 0.01; *** *p* < 0.001.

## Electronic supplementary material

Below is the link to the electronic supplementary material.

Supplementary Material 1

## Data Availability

The datasets used and/or analyzed during the current study available from the corresponding author on reasonable request.

## Abbreviations

KLF: Kruppel-like factor
KLF15: Kruppel-like factor 15
GABA: γ-aminobutyrate
ACh: Acetylcholine
AAV: Adeno-associated virus
Glu: Glutamic acid
RIPA: Radioimmunoprecipitation assay lysis buffer
SDS-PAGE: Polyacrylamide gel electrophoresis
ECL: Enhanced chemiluminescence
PFA: Polyformaldehyde
PBS: Phosphate buffered saline
DAPI: 4,6-diamidino-2-phenylindole
