# Supplementary material for: Distribution and functional significance of KLF15 in mouse cerebellum
Source: Mol Brain. 2025 Jan 21;18:3. doi: 10.1186/s13041-025-01172-3 (PMC11749119; doi:10.1186/s13041-025-01172-3)
Supplement: Supplementary file 1 — Supplementary Material 1 [file 13041_2025_1172_MOESM1_ESM.docx]

**
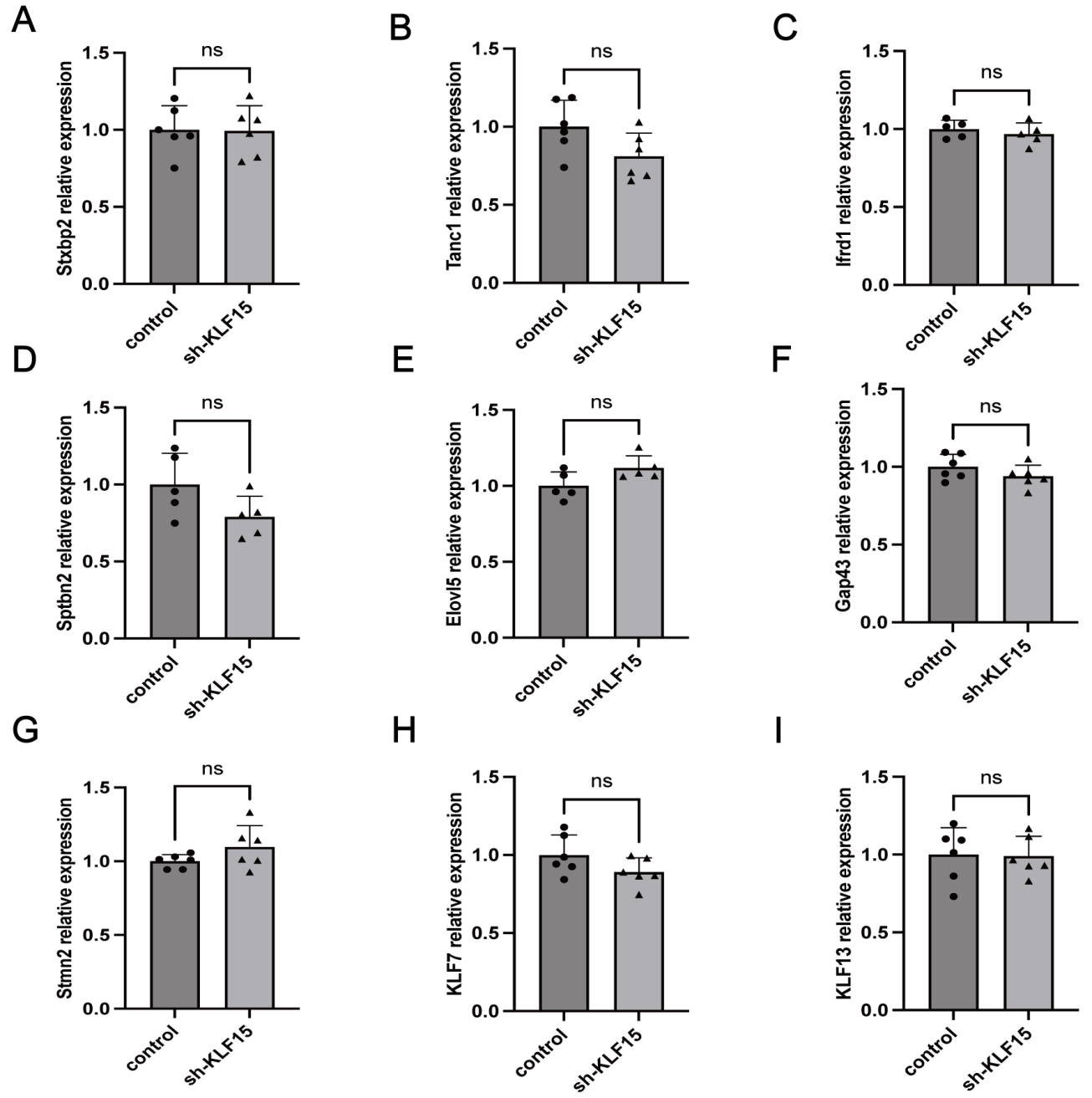
**

**Additional Figure 1:** The injection of the virus does not affect the expression of other genes. **A** Relative expression of Stxbp2 genes. **B** Relative expression of Tanc1 genes. **C** Relative expression of Ifrd1 genes. **D** Relative expression of Sptbn2 genes. **E** Relative expression of Elovl5 genes. **F** Relative expression of Gap43 genes. **G** Relative expression of Stmn2 genes. **H** Relative expression of KLF7 genes. **I** Relative expression of KLF13 genes. Data are shown as the mean ± SD, ns, no significant, vs. control group (two-tailed Student’s t-test).

**
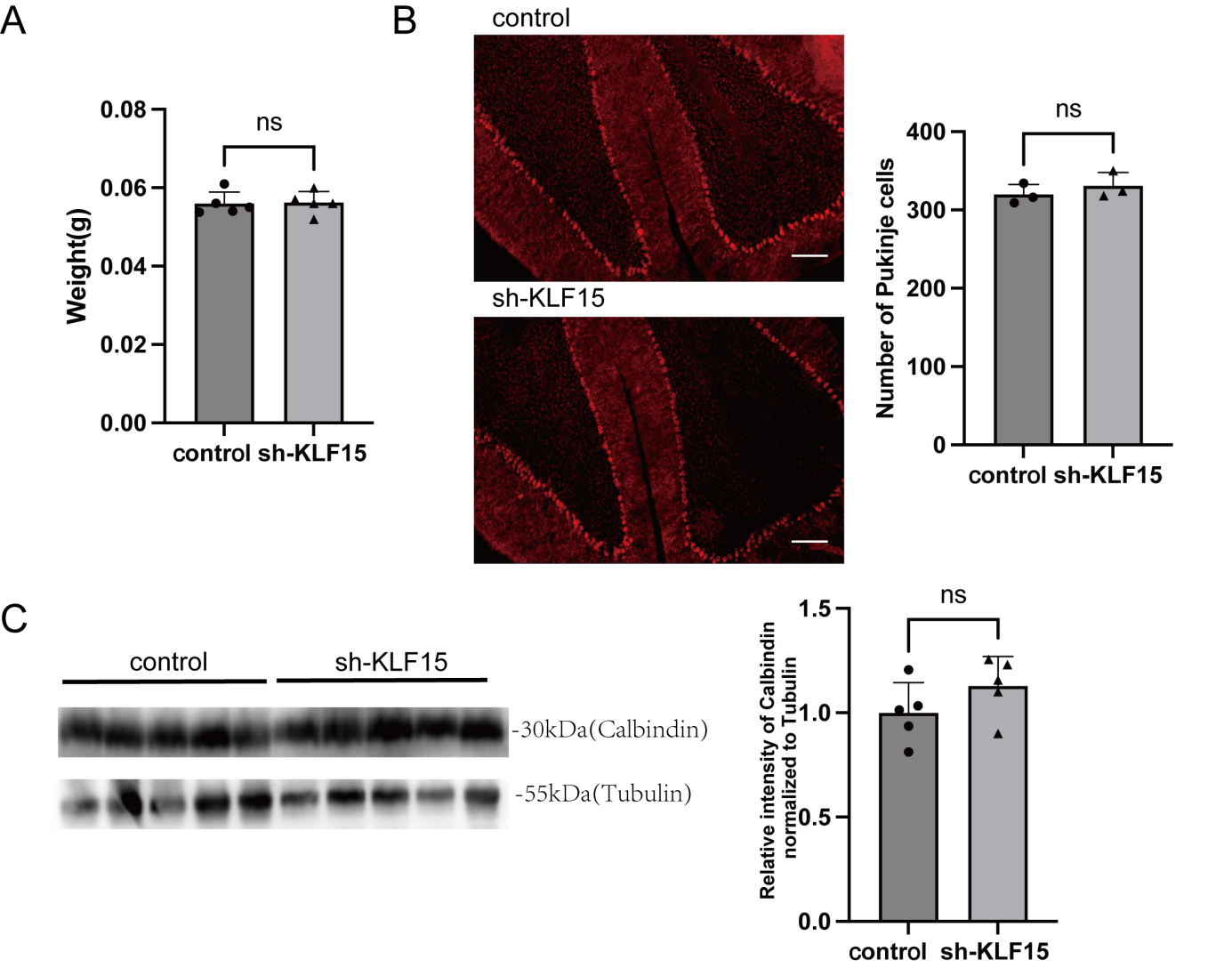
**

**Additional Figure 2:** Changes in Purkinje cell count after knocking down KLF15 in the cerebellum of adult mice. **A** Weighting the cerebellum of control mice and KLF15 knockdown mice. **B** Immunofluorescence staining of cerebellum in the control mice and sh-KLF15 mice. On the right is the quantitative statistics of Purkinje cell number. Scale bar = 200 μm. **C** Western blot and grayscale analysis of calbindin in the cerebellum of control mice and KLF15 knockdown mice. Data are shown as the mean ± SD, ns, no significant, vs. control group (two-tailed Student’s t-test).
